# A Challenging Diagnosis of Laryngeal Neuroendocrine Tumor (NET): Final Classification as NET Grade 3 After Total Laryngectomy

**DOI:** 10.7759/cureus.90729

**Published:** 2025-08-22

**Authors:** Naoya Sanda, Nayuta Tsushima, Hayato Imanari, Akihiro Homma

**Affiliations:** 1 Department of Otolaryngology – Head and Neck Surgery, Faculty of Medicine and Graduate School of Medicine, Hokkaido University, Sapporo, JPN

**Keywords:** cancer of the head and neck, laryngeal tumors, neuroendocrine carcinoma (nec), neuroendocrine neoplasm, neuroendocrine tumors (net), total laryngectomy

## Abstract

Laryngeal neuroendocrine neoplasms (NENs) are rare tumors that account for a small proportion of all laryngeal malignancies. They exhibit a wide range of differentiation and proliferative activity, and are often graded accordingly, using markers such as mitotic count and Ki-67 labeling index. We present the case of a 76-year-old man with progressive dysphagia and a supraglottic mass. Initial biopsy findings were inconclusive, raising suspicion of poorly differentiated carcinoma or metastatic disease. A re-biopsy of the larynx under general anesthesia and neck dissection revealed features consistent with neuroendocrine tumor (NET) Grade 2 (Ki-67 index 17%). Due to the limited efficacy of nonsurgical treatments and absence of somatostatin receptor expression, total laryngectomy was performed. Histopathological analysis of the resected specimen demonstrated increased mitotic activity and a Ki-67 index of 28%, leading to a revised diagnosis of NET Grade 3. This case highlights the diagnostic limitations of small biopsy specimens in the head and neck region and underscores the importance of stepwise evaluation, including surgical pathology, in establishing an accurate diagnosis and guiding curative treatment decisions in rare laryngeal NETs.

## Introduction

Most malignant tumors of the larynx are squamous cell carcinomas, with other histological subtypes being relatively uncommon [1]. Among these, neuroendocrine neoplasms (NENs) of the larynx are exceedingly rare, accounting for less than 1% of all laryngeal malignancies [2]. NENs are defined as epithelial tumors with neuroendocrine differentiation that can arise in a variety of organs [3]. The most common primary sites are the gastrointestinal tract (over 60%) and the lungs (over 20%) [4]. In contrast, NENs arising from other sites, such as the head and neck region, thymus, thyroid, breast, skin, and genitourinary tract, are considered relatively rare [5].　

According to the 2022 World Health Organization (WHO) classification of head and neck tumors, laryngeal NENs are categorized into well-differentiated neuroendocrine tumors (NETs), including typical and atypical carcinoids, and poorly differentiated neuroendocrine carcinomas (NECs), comprising small cell and large cell subtypes [6]. This classification follows the unified International Agency for Research on Cancer (IARC)/WHO framework and is based on pathological features such as the Ki-67 labeling index, mitotic rate per 2 mm², and the presence or absence of necrosis, key indicators of tumor proliferative activity and aggressiveness that guide prognosis and treatment [7]. However, accurate diagnosis can be challenging when only limited biopsy samples are available.

Herein, we report a rare case of laryngeal NEN that underwent multiple histopathological evaluations and was ultimately reclassified as Grade 3 NET following total laryngectomy.

## Case presentation

A 76-year-old man presented with progressive dysphagia. During an upper gastrointestinal endoscopy performed at a local hospital, a tumor-like lesion was incidentally noted in the larynx, prompting referral to the otolaryngology department for further evaluation. Flexible laryngoscopy was performed and revealed a mass lesion in the supraglottic region. The initial biopsy of the lesion did not show features suggestive of squamous cell carcinoma. Instead, the histological findings raised suspicion of a poorly differentiated carcinoma, possibly metastatic from the lung or thyroid. Imaging studies also revealed enlargement of two cervical lymph nodes on the left side.　

The patient was referred to our department for further diagnostic workup and treatment planning. His medical history included hypertension, hyperlipidemia, and borderline diabetes mellitus, all of which were managed with oral medications. He had a smoking history of 30 cigarettes per day for 35 years and occasional alcohol use. There was no family history of head and neck cancer, though his younger brother had lung cancer. The patient's performance status was 0 according to the Eastern Cooperative Oncology Group (ECOG) criteria.

**Figure 1:**
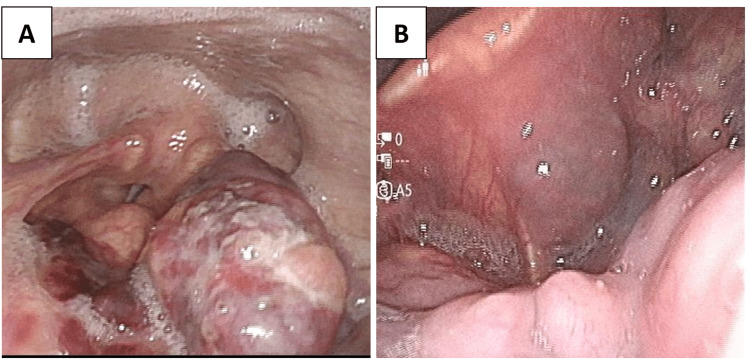
Endoscopic findings of the epiglottic lesion (A) Flexible laryngoscopy reveals a dark reddish, irregular, elevated mass on the laryngeal surface of the epiglottis. There is no extension to the pharyngeal mucosa, no vocal cord paralysis, and the upper airway remains patent. (B) A mass with cystic features is observed in the vallecula (the space between the base of the tongue and the epiglottis), which appears to be contiguous with the lesion shown in (A). The cystic component could mimic a benign condition such as an epiglottic cyst, potentially obscuring the suspicion of malignancy.

**Figure 2:**
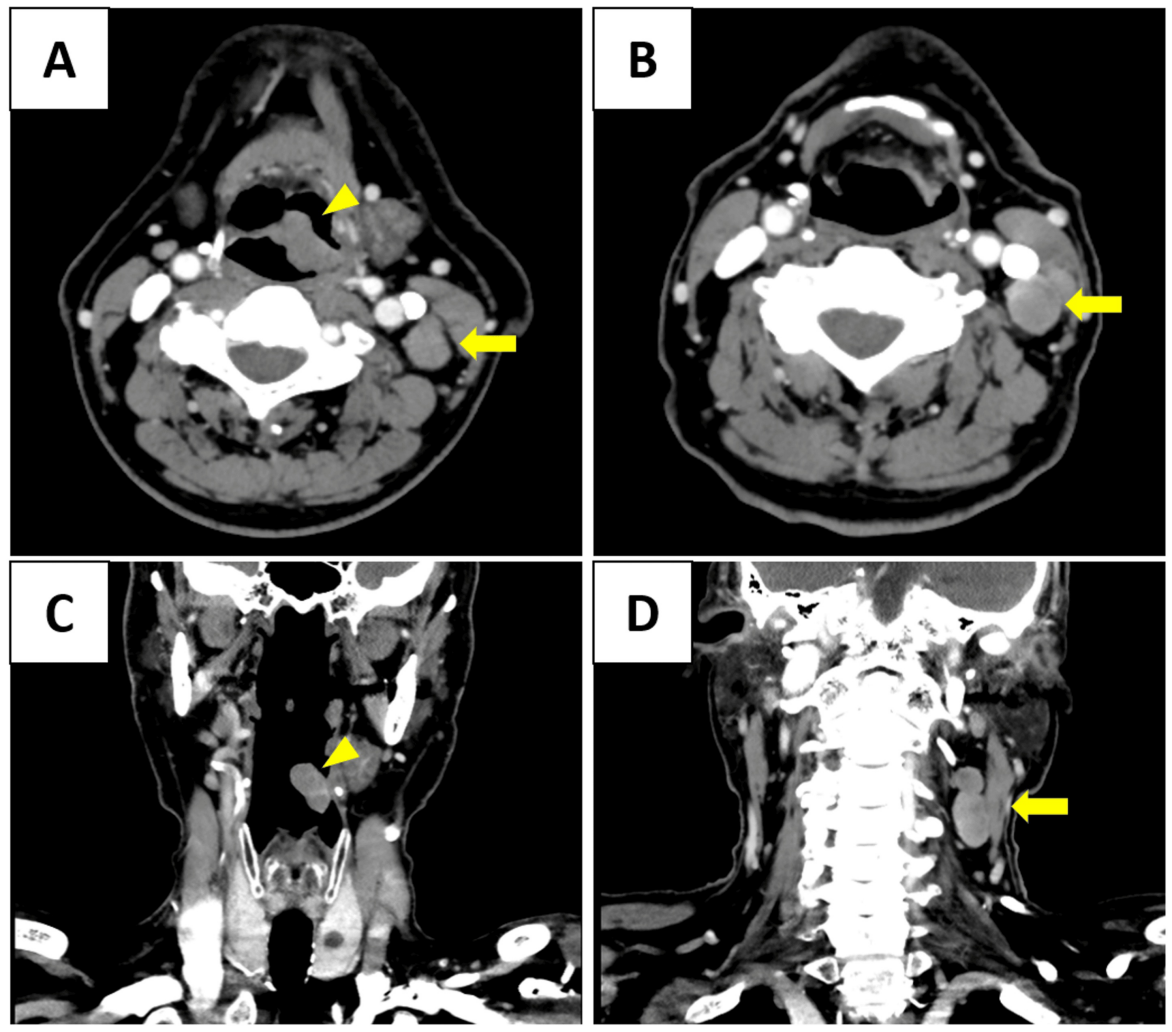
Contrast-enhanced computed tomography (CT) findings (A, B) Axial and (C, D) coronal contrast-enhanced CT images reveal a soft tissue mass measuring 18 mm × 12 mm with mild enhancement in the left supraglottic region (yellow arrowheads), along with multiple enlarged lymph nodes in the left internal jugular chain, the largest measuring 18 mm × 18 mm (yellow arrows). The imaging features are not specific and could be seen in other supraglottic tumors such as squamous cell carcinoma, making histopathological confirmation essential.

At our institution, flexible laryngoscopy was performed again and revealed an irregularly elevated lesion measuring 18 mm × 12 mm on the left laryngeal surface of the epiglottis (Figure 1). Contrast-enhanced computed tomography (CT) demonstrated irregular wall thickening with mild enhancement on the left side of the epiglottis (Figure 2A). Multiple enlarged lymph nodes were noted in the left internal deep cervical region, with the largest measuring 18 mm × 18 mm (Figure 2B).

**Figure 3:**
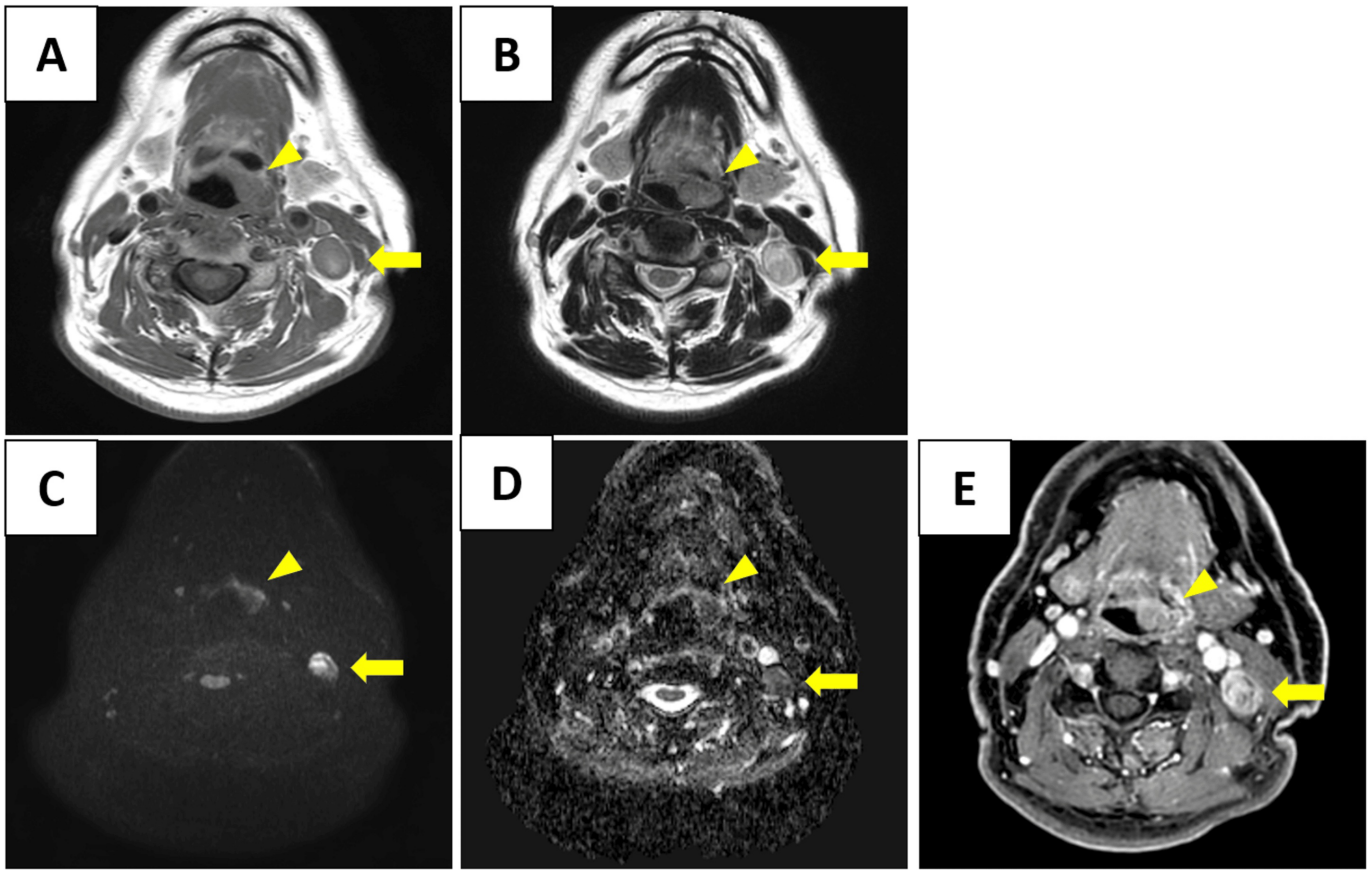
Magnetic resonance imaging (MRI) findings (all axial sections) (A) T1-weighted image, (B) T2-weighted image, (C) diffusion-weighted image (DWI), (D) apparent diffusion coefficient (ADC) map, (E) contrast-enhanced T1-weighted image with gadolinium. An irregular mass measuring 18 × 12 mm is observed on the left side of the epiglottis, showing iso-intensity on both T1- and T2-weighted images (yellow arrowheads). Enlarged lymph nodes are also noted in the left internal jugular region (yellow arrows). The epiglottic tumor and cervical lymph nodes appear hyperintense on DWI and hypointense on the ADC map, indicating restricted diffusion. Both lesions exhibit contrast enhancement following gadolinium administration. Similar to CT, these MRI findings are not pathognomonic and could overlap with those of more common supraglottic malignancies such as squamous cell carcinoma, underscoring the need for tissue diagnosis.

Magnetic resonance imaging (MRI) showed an irregular mass measuring 18 × 12 mm on the left side of the epiglottis, with iso-intensity on both T1- and T2-weighted images. No apparent invasion of the pre-epiglottic space, aryepiglottic fold, or lateral pharyngeal wall was observed (Figure 3).

**Figure 4:**
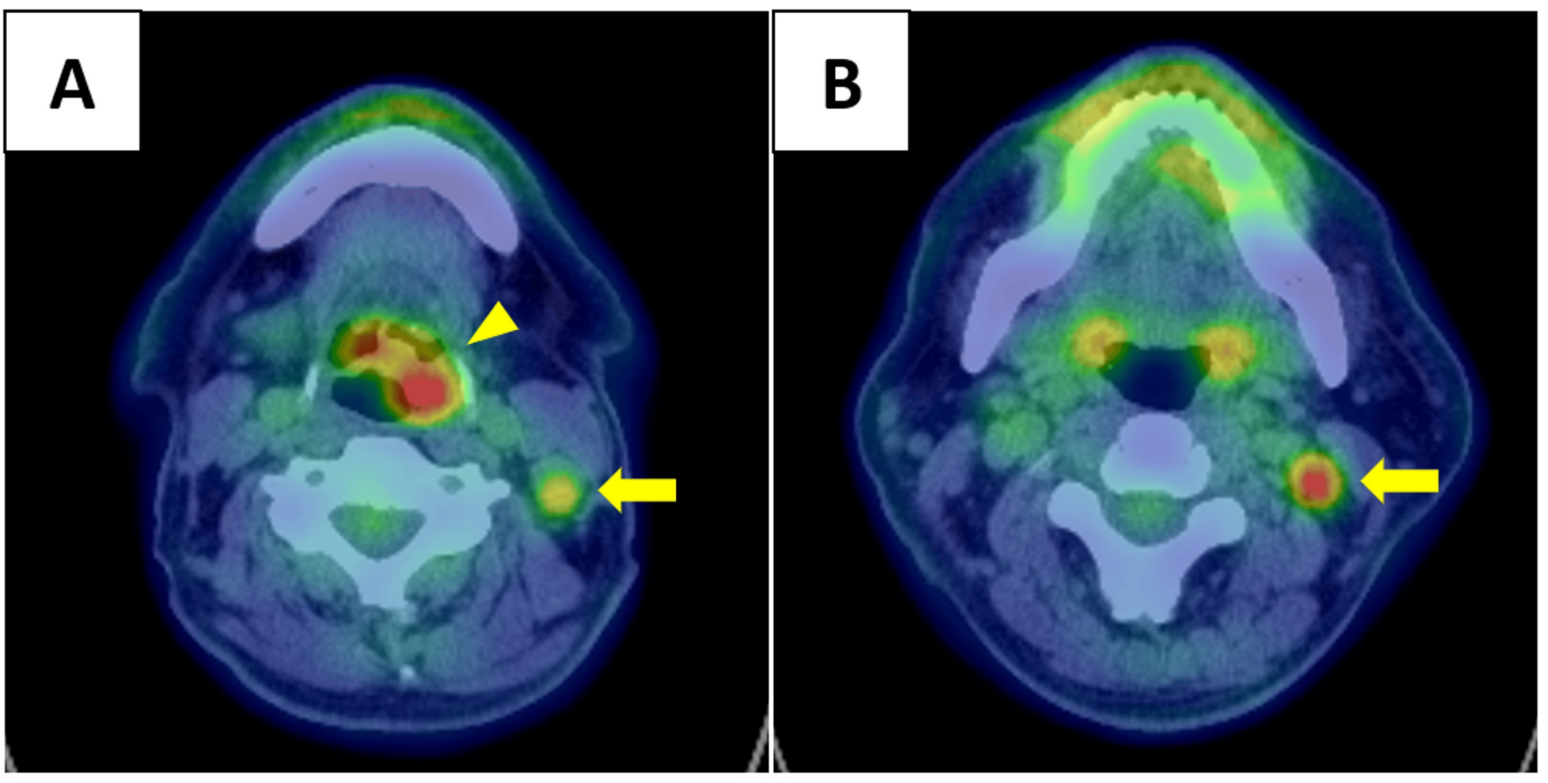
Axial view of positron emission tomography-computed tomography (PET-CT) Increased fluorodeoxyglucose (FDG) uptake is observed in the lesion on the left side of the epiglottis (yellow arrowhead) and in multiple lymph nodes in the left cervical region (yellow arrows), with a maximum standardized uptake value (SUVmax) of 8.1. No distant metastasis is identified.

Positron emission tomography-CT (PET-CT) demonstrated increased fluorodeoxyglucose (FDG) uptake in both the supraglottic tumor and the left cervical lymph nodes, with a maximum standardized uptake value (SUVmax) of 8.1 (Figure 4). No distant metastasis was identified.

No primary tumor was found in the lungs or thyroid. At this stage, surgical resection was not primarily considered, as the presumptive diagnosis of a poorly differentiated carcinoma of laryngeal origin suggested that the disease could be adequately controlled with chemoradiotherapy. However, re-evaluation of the biopsy specimen at our institution suggested a NET of laryngeal origin or a metastatic medullary thyroid carcinoma.

To establish a definitive diagnosis, we performed a laryngeal biopsy under general anesthesia along with a left neck dissection. Prior to this procedure, fine-needle aspiration cytology of the left cervical lymph nodes had suggested metastatic medullary thyroid carcinoma, raising the possibility that the histopathology of the laryngeal lesion and the cervical lymph nodes might differ (i.e., multiple primary tumors). Therefore, the combined procedure under general anesthesia allowed both diagnostic confirmation and therapeutic management in a single session. This treatment plan was determined through multidisciplinary discussion involving the departments of otolaryngology, medical oncology, and radiology.

**Figure 5:**
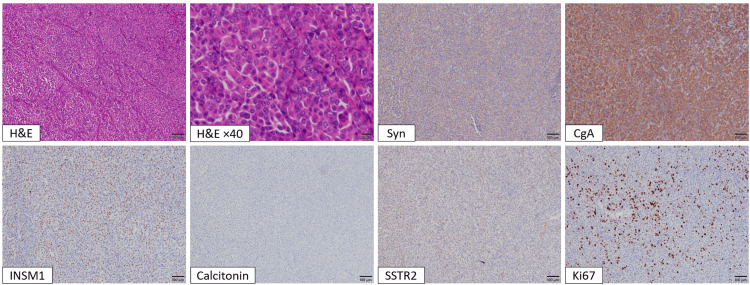
Histopathological and immunohistochemical findings of the laryngeal neuroendocrine tumor from the second laryngeal biopsy performed under general anesthesia All panels are captured at 100× magnification using a 10× objective lens, except for the hematoxylin and eosin (H&E) ×40 panel, which is captured at 400× magnification using a 40× objective lens. H&E staining shows atypical cells with salt-and-pepper–like round nuclei and eosinophilic cytoplasm arranged in trabecular and sheet-like patterns. Immunohistochemically, the tumor cells are positive for synaptophysin (Syn), chromogranin A (CgA), and INSM1, and negative for calcitonin and somatostatin receptor subtype 2 (SSTR2). The Ki-67 labeling index shows regional variability, with a hotspot value of 17%.

**Figure 6:**
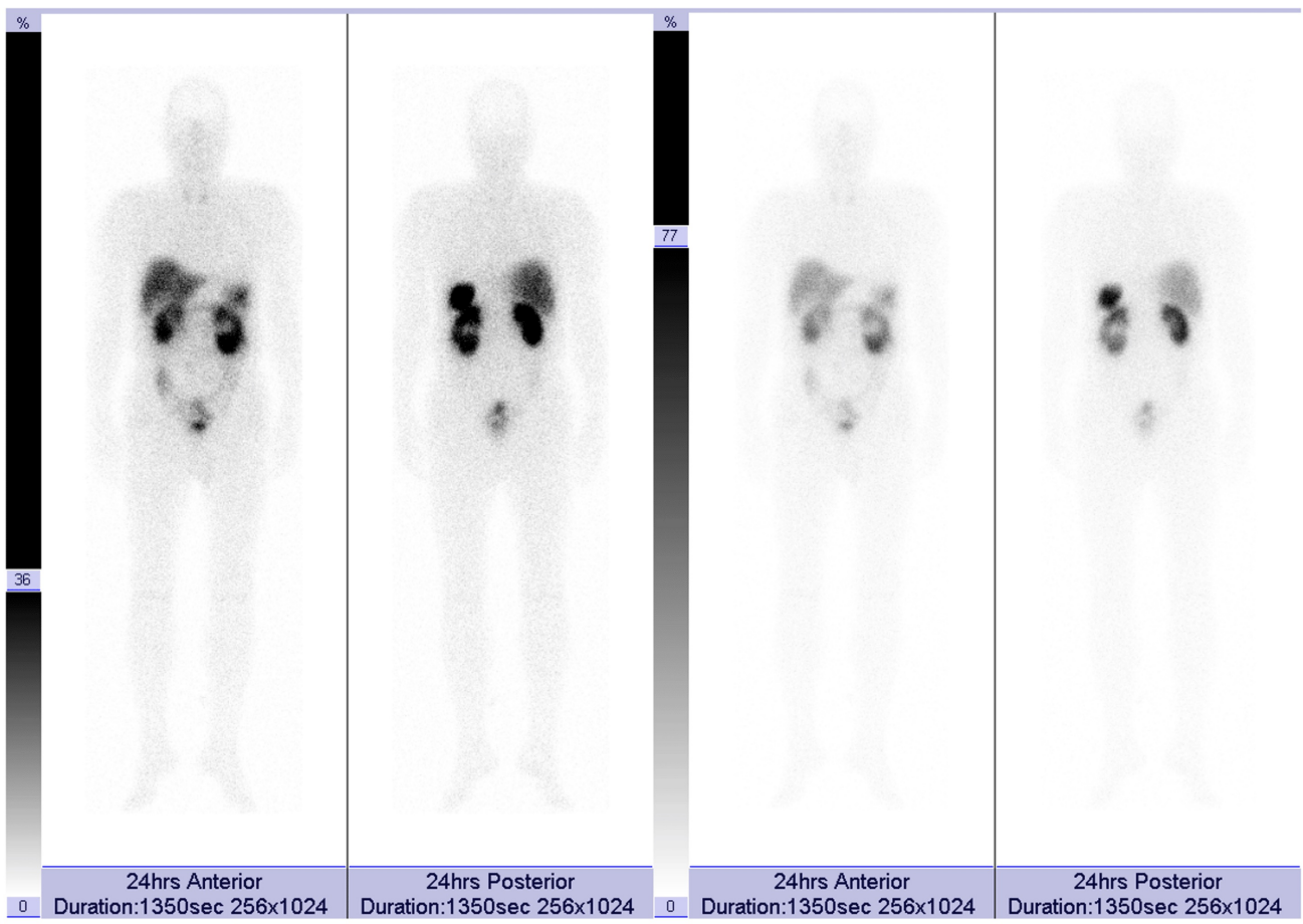
Octreotide scintigraphy (Octreoscan) No radiotracer uptake is observed in the laryngeal lesion, indicating absence of somatostatin receptor expression.

Histopathological examination confirmed the diagnosis of a NET of the larynx, corresponding to Grade 2. Of the 23 resected cervical lymph nodes, 14 showed metastatic involvement, and the morphology and immunophenotype were consistent with the primary laryngeal lesion, supporting a single primary origin. Immunohistochemical staining was positive for synaptophysin, chromogranin A, and INSM1, but negative for p63 and calcitonin. The Ki-67 labeling index was 17% in the hotspot area (Figure 5). Immunostaining for somatostatin receptor subtype 2 (SSTR2) was negative (score 0), and preoperative octreotide scintigraphy (Octreoscan) showed no tracer uptake in the laryngeal lesion (Figure 6).

Following confirmation of the diagnosis through the laryngeal biopsy and left cervical lymph node dissection, which established the lesion as a rare primary NET of the larynx, a multidisciplinary tumor board meeting was held with specialists in gastrointestinal surgery, radiation oncology, medical oncology, and nuclear medicine. The tumor was considered unsuitable for somatostatin analog therapy or peptide receptor radionuclide therapy (PRRT), and radiation therapy was expected to have limited efficacy. Curative resection was therefore recommended.

**Figure 7:**
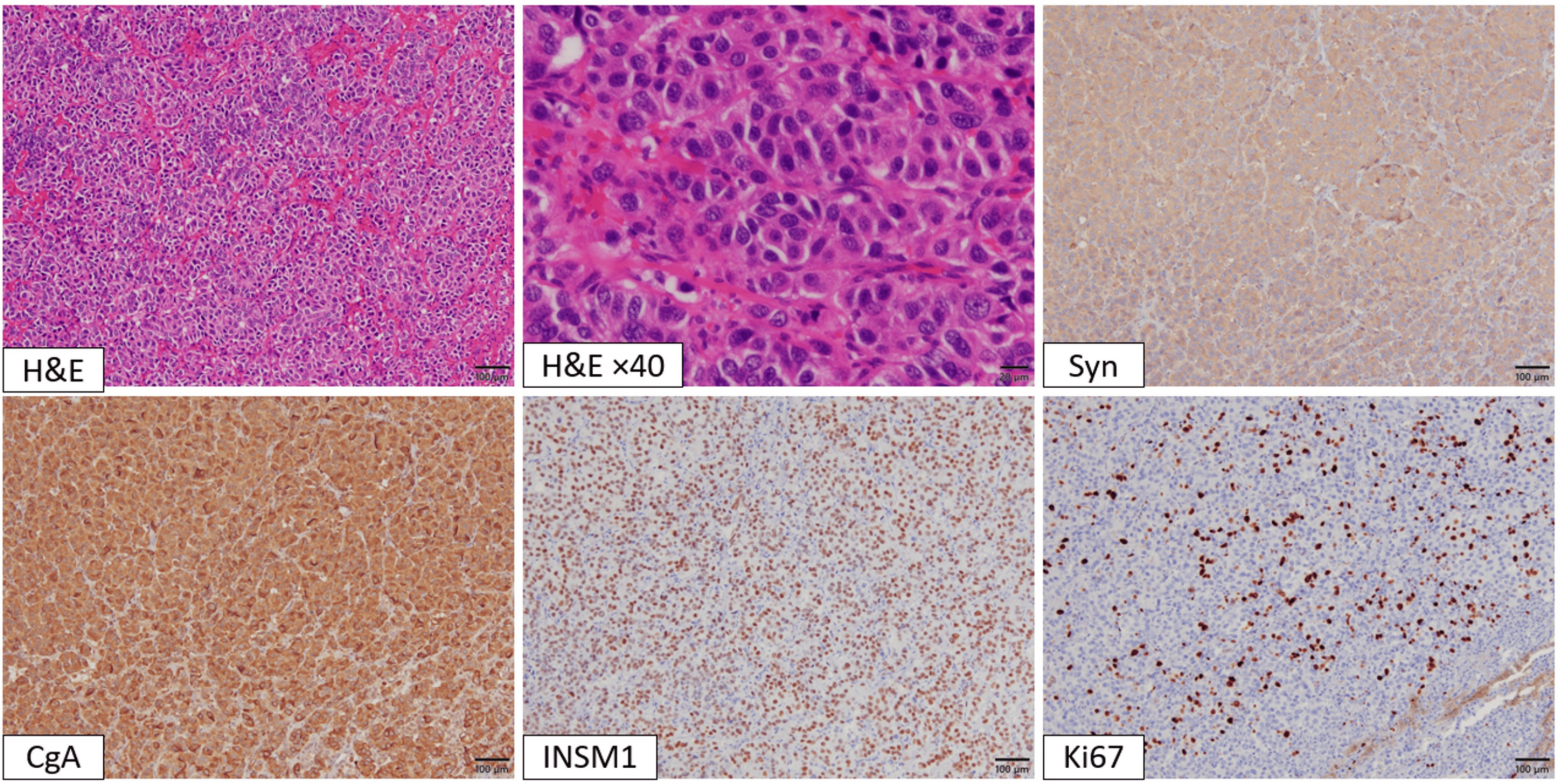
Histopathological and immunohistochemical findings of the resected laryngeal neuroendocrine tumor following total laryngectomy All panels are captured at 100× magnification using a 10× objective lens, except for the hematoxylin and eosin (H&E) ×40 panel, which is captured at 400× magnification using a 40× objective lens. H&E staining shows marked nuclear pleomorphism and a trabecular-to-solid growth pattern. Immunohistochemically, the tumor cells are positive for synaptophysin (Syn), chromogranin A (CgA), and INSM1. The Ki-67 labeling index is elevated in areas with high-grade features, reaching 28% in the hotspot region.

The patient subsequently underwent total laryngectomy. Prophylactic dissection of the contralateral (right) cervical lymph nodes was omitted, as no enlarged nodes were identified, and the clinical benefit of such a procedure was deemed low. Histopathological examination of the resected specimen revealed pronounced nuclear atypia, increased mitotic activity, and a Ki-67 labeling index of 28%, leading to reclassification of the tumor as consistent with Grade 3 NET. Immunohistochemically, the tumor was positive for AE1/AE3, synaptophysin, chromogranin A, and INSM1, and showed weak-to-moderate positivity for p53 (Figure 7). The surgical margins were free of tumor cells.

Based on the negative margins and absence of distant metastases, no adjuvant therapy, such as postoperative radiotherapy or chemoradiotherapy, was administered. At the three-month follow-up, the patient remains recurrence-free.

## Discussion

We encountered a rare case of laryngeal NET that was ultimately diagnosed as Grade 3 following total laryngectomy. Initial and even re-biopsy results were inconclusive or underestimated the grade, highlighting the diagnostic challenges and importance of stepwise evaluation for such tumors.

Histologically, NENs are characterized by round-to-oval or spindle-shaped cells with granular or pale eosinophilic cytoplasm arranged in nests, trabeculae, or sheets [8]. Immunohistochemically, markers such as synaptophysin, chromogranin A, INSM1, and Ki-67 are essential for evaluation. Among these, synaptophysin is regarded as the most sensitive marker, whereas chromogranin A is considered the most specific [9,10]. INSM1 has emerged as a useful adjunct marker, with a reported sensitivity of 99% for head and neck NENs [11,12].

Although the Ki-67 proliferation index is a key marker for classification, most head and neck NETs tend to exhibit a Ki-67 index below 20% [13,14]. While high-grade NETs (NET G3) in the head and neck region have not been clearly defined, NECs are generally characterized by a markedly elevated Ki-67 index, often exceeding 55% [13,14].

In the present case, the second biopsy demonstrated a Ki-67 index of 17%, initially interpreted as consistent with a lower-grade NET. In the resected specimen, the Ki-67 index increased to 28%, reflecting higher proliferative activity than suggested by the biopsy. This change is clinically important because it provides a more accurate assessment of tumor aggressiveness, which is essential for guiding diagnostic reassessment and ensuring appropriate therapeutic planning, even if the initial biopsy appears borderline.

Aberrant p53 expression and loss of retinoblastoma (Rb) protein are significantly more frequent in NECs [13,15,16]. In a large cohort study, abnormal p53 immunostaining was observed in 91.5% of NECs compared to only 12.7% of NET G3s, while Rb protein loss occurred in 68.3% of NECs but only 10.6% of NET G3s [16]. According to the 2022 WHO classification, head and neck NETs typically do not show p53 overexpression or Rb protein loss [6].

In the present case, the tumor demonstrated a Ki-67 index of 28%, which is below the typical threshold for NEC. Immunohistochemical analysis revealed only weak-to-moderate p53 positivity without Rb protein loss. These findings are more consistent with a diagnosis of NET G3 than NEC. In a meta-analysis of 436 laryngeal NENs by van der Laan et al., 5% were typical carcinoids (NET G1), 37% atypical carcinoids (NET G2 or G3), and 49% NECs [7]. A retrospective study of 27 patients by Bal et al., nine cases had NET G2 and 18 had NEC, indicating that NET G2-G3 may be more common than NET G1 in the larynx [14].

Clinically, NET G2-G3 tends to occur in males (70%) with a median age of 63 years and a strong history of smoking (94%) [7]. Most tumors arise in the supraglottic region. The demographic details and tumor location in our patient are consistent with these findings. Macroscopically, laryngeal NETs are typically observed as dark red, smooth-surfaced polypoid or nodular masses and are often mistaken for benign vascular lesions such as hemangiomas [17,18]. This appearance is distinct from the cauliflower-like or ulcerative growth pattern commonly seen in squamous cell carcinomas of the larynx. In our case, the tumor presented both cystic and elevated components and was ultimately diagnosed as NET.

Regarding treatment and prognosis, van der Laan et al. reported that the five-year disease-specific survival (DSS) for NET G2-G3 was higher in the surgery group (60.2%) than in the radiation therapy group (53.8%) (p = 0.035). Conversely, in NEC cases, patients treated with chemoradiotherapy had significantly better outcomes (DSS: 30.8%) than those receiving other treatments (12.9%). These findings support surgery as the treatment of choice for resectable laryngeal NET G2-G3 [7].

Somatostatin receptor subtype 2 (SSTR2) expression is used to determine eligibility for somatostatin analog therapy and peptide receptor radionuclide therapy (PRRT) [16]. In this case, both SSTR2 immunostaining and octreotide scintigraphy were negative, indicating that these therapies were not appropriate.

The initial diagnosis of NENs is often challenging due to their rarity and histological overlap with poorly differentiated adenocarcinomas or metastatic tumors [18,19]. Several reports have described diagnostic revisions following surgical resection (Table 1) [18-20]. In our case, the tumor was initially suspected to be a poorly differentiated carcinoma, but subsequent histopathological evaluation and multidisciplinary review led to a final diagnosis of NET G3. Similar diagnostic challenges were noted in previous reports; Yang et al. described a case initially diagnosed as atypical cell hyperplasia on biopsy, with the final diagnosis of NET G2 made only after laryngectomy [18], while Wang et al. reported a case initially diagnosed as poorly differentiated adenocarcinoma, later revised to NET G3 after definitive surgery [19]. These patterns illustrate that preoperative biopsies may underestimate or misclassify laryngeal NENs, particularly when the specimen is limited or the tumor shows overlapping histological features. Our case, consistent with these reports, emphasizes the need to include NENs in the differential diagnosis of supraglottic tumors with atypical histology and to pursue thorough histopathological confirmation when suspicion remains.

**Table 1:** Comparison of well-differentiated laryngeal NET cases NET, neuroendocrine tumor; ND, neck dissection; CRT, chemoradiotherapy; ANED, alive no evidence of disease; DOD, died of disease *Clinical staging was based on the 8th edition of the UICC classification [21].

Case	Author (Year)	Age	Sex	Tumor Site	Clinical Stage*	Biopsy Diagnosis (before initial treatment)	Final Postoperative Diagnosis	Treatment	First Recurrence	Outcome (Follow-up)
											1	Present case	76	M	Epiglottis	T1N2bM0	poorly differentiated carcinoma	NET G3	Total laryngectomy+ND	-	ANED (3 months)
2	Yang et al. (2024) [18]	67	M	Epiglottis	unknown	atypical cell hyperplasia	NET G2	Laryngectomy+ND	-	ANED (12 months)
3	Wang et al. (2024) [19]	67	M	Epiglottis	T3N2bM0	poorly differentiated adenocarcinoma	NET G3	Surgery (horizontal hemilaryngectomy+ND) + CRT	-	ANED (12 months)
4	Kashima et al.(2024) [20]	74	M	Epiglottis	T1N0M0	neuroendocrine tumor	NET G2	Horizontal partial laryngectomy	Gastrointestinal recurrence (41 months)	DOD (41 months)

Because biopsy samples in the head and neck region are often limited, establishing a definitive diagnosis at the initial presentation is particularly difficult for NETs, whose treatment and prognosis depend on tumor grade. Accurate classification requires comprehensive histopathological and immunohistochemical analysis, making a stepwise diagnostic approach essential. In cases with inconclusive or atypical findings, repeated or more extensive biopsies should be performed to secure sufficient tissue before curative treatment is determined.

To ensure accurate staging and guide optimal treatment, neck dissection was performed prior to total laryngectomy in the present case. Fine-needle aspiration cytology of the left cervical lymph nodes had suggested metastatic medullary thyroid carcinoma, raising the possibility that the histopathology of the laryngeal lesion and the cervical lymph nodes might differ (i.e., multiple primary tumors). Performing the combined procedure under general anesthesia allowed both diagnostic confirmation and therapeutic management in a single session. This strategy was determined through multidisciplinary discussion involving otolaryngology, medical oncology, and radiology, reflecting a careful balance between diagnostic uncertainty and the need for comprehensive, curative treatment. Prophylactic dissection of the contralateral (right) cervical lymph nodes was not performed, as no enlarged nodes were identified, and the potential clinical benefit of such a procedure was considered low. Moreover, even if delayed nodal metastasis were to occur, salvage surgery was deemed feasible, and the omission of right neck dissection was preferred in order to avoid potential adverse events such as shoulder dysfunction.

Overall, these management decisions illustrate the practical importance of individualized, stepwise planning in laryngeal NETs, ensuring that both diagnostic accuracy and curative treatment objectives are met while minimizing unnecessary interventions.

Given that the surgical margins were negative and no distant metastases were detected, no adjuvant therapy was administered. However, NET Grade 3 has been associated with a risk of recurrence and distant metastasis [7,20], emphasizing the need for long-term and systemic follow-up. In our case, the follow-up period was relatively short (three months), which is a limitation in fully assessing the prognosis. Longer-term follow-up data are needed to better understand the risk of recurrence and metastasis in such cases.

## Conclusions

NENs of the larynx are rare and diagnostically challenging, particularly when biopsy specimens are limited. This case demonstrates that reliance on initial histopathological findings can lead to underestimation of tumor grade, potentially influencing treatment selection. A stepwise approach, including re-biopsy, surgical pathology, and multidisciplinary evaluation, is essential for accurate diagnosis and optimal management. In this patient, the decision to perform neck dissection prior to total laryngectomy and to prioritize surgery was guided not solely by staging considerations, but by the possibility of multiple primary tumors and the need for stepwise diagnostic and therapeutic evaluation. Although the follow-up period was short (three months), limiting long-term prognostic assessment, the case highlights the importance of carefully reassessing diagnostic findings and tailoring management according to both tumor characteristics and sequential clinical information. This experience provides practical guidance for clinicians in approaching complex laryngeal NETs.
